# Sequence variation and functional analysis of a *FRIGIDA* orthologue (*BnaA3.FRI*) in *Brassica napus*

**DOI:** 10.1186/s12870-018-1253-1

**Published:** 2018-02-13

**Authors:** Licong Yi, Chunhong Chen, Shuai Yin, Haitao Li, Zhaohong Li, Bo Wang, Graham J. King, Jing Wang, Kede Liu

**Affiliations:** 1 National Key Laboratory of Crop Genetic Improvement, College of Plant Science and Technology, Huazhong Agricultural University, Wuhan, Hubei 430070 China; 2 Southern Cross Plant Science, Southern Cross University, Lismore, NSW 2480 Australia

**Keywords:** *Brassica napus*, *FRIGIDA*, Haplotype, Flowering time, Crop type

## Abstract

**Background:**

Allelic variation at the *FRIGIDA* (*FRI*) locus is a major contributor to natural variation of flowering time and vernalization requirement in *Arabidopsis thaliana*. Dominant *FRI* inhibits flowering by activating the expression of the MADS box transcriptional repressor *FLOWERING LOCUS C* (*FLC*), which represses flowering prior to vernalization. Four *FRI* orthologues had been identified in the domesticated amphidiploid *Brassica napus*. Linkage and association studies had revealed that one of the *FRI* orthologues, *BnaA3.FRI*, contributes to flowering time variation and crop type differentiation.

**Results:**

Sequence analyses indicated that three out of the four *BnaFRI* paralogues, *BnaA3.FRI*, *BnaA10.FRI* and *BnaC3.FRI*, contained a large number of polymorphic sites. Haplotype analysis in a panel of 174 *B. napus* accessions using PCR markers showed that all the three paralogues had a biased distribution of haplotypes in winter type oilseed rape (*P* < 0.01). Association analysis indicated that only *BnaA3.FRI* contributes to flowering time variation in *B. napus*. In addition, transgenic functional complementation demonstrated that mutations in the coding sequence of *BnaA3.FRI* lead to weak alleles, and subsequently to flowering time variation.

**Conclusion:**

This study for the first time provides a molecular basis for flowering time control by *BnaA3.FRI* in *B. napus*, and will facilitate predictive oilseed rape breeding to select varieties with favorable flowering time and better adaption to latitude and seasonal shifts due to changing climate.

**Electronic supplementary material:**

The online version of this article (10.1186/s12870-018-1253-1) contains supplementary material, which is available to authorized users.

## Background

Timely transition from vegetative to reproductive growth is of great significance for plants, in avoiding adverse environments and ensuring seed set. In the model species *Arabidopsis thaliana*, a flowering time regulatory gene network has been established [1]. External and internal signals associated with different pathways converge on the common set of key integrators *FLOWERING LOCUS T* (*FT*), *LEAFY* (*LFY*), and *SUPPRESSOR OF OVEREXPRESSION OF CONSTANS* (*SOC1*), which act on downstream genes to promote floral organ formation [1–4]. *FT* is a key flowering integrator gene encoding a florigen protein that moves through the phloem from leaves to the shoot apex, and induces the floral transition in many plant species [5, 6].

The photoperiod and vernalization pathways are the two major pathways responding to environmental cues that determine the flowering time of *A. thaliana,* as well as most other plants [7]. Based on vernalization requirement, genotypes of *A. thaliana* may be grouped into late flowering (winter-annual) and early flowering ecotypes (summer-annual). *FLOWERING LOCUS C* (*FLC*) and *FRIGIDA* (*FRI*) are the two key genes in the vernalization pathway [8–10]. Dominant *FRI* represses flowering through activating the expression of *FLC* [11], which encodes a MADS box transcriptional regulator and represses flowering through directly inhibiting the expression of *SOC1* gene [8, 12]. Vernalization promotes flowering through proteasome-mediated degradation of FRI protein and epigenetic silencing of *FLC* [13, 14]. Allelic variation at either *FRI* or *FLC* locus will lead to a summer-annual life cycle of *A. thaliana* [10]. Loss-of-function mutations of *FRI* were demonstrated to be the major determinant of vernalization requirement and explain most flowering time variation of early-flowering ecotypes [15, 16].

*Brassica napus* (AC genome, oilseed rape, canola or rapeseed) is the third most important oil crop in the world. This domesticated amphidiploid species most likely originated from a natural cross between *B. rapa* (A genome) and *B. oleracea* (C genome) ~ 7500 years ago [17]*.* As with *Arabidopsis*, *B. napus* can have a vernalization requirement to initiate flowering. Based on vernalization requirement, the three crop types of oilseed rape recognized are spring-type (SOR), semi-winter type (SWOR, rapeseed accessions that initiate flowering with a moderate vernalization condition (0 - 4 °C for 15-30 d)) and winter-type (WOR). Due to historical duplication events that have occurred since *Arabidopsis* and *Brassica* diverged from a common ancestor, the diploid genomes of *B. rapa* and *B. oleracea* appear triplicated compared with *Arabidopsis*, and so the *B. napus* crop genome is extreme complex [17]. A large number of QTLs and candidate genes that contribute to flowering time variation have been documented in *B. napus* [18–23]. Orthologues of major flowering genes such as *CONSTANS* (*CO*), *FLC*, and *FT* have been found to be functionally conserved between *B. napus* and *Arabidopsis*. For example, the *BnaFLC* orthologues were proven to confer winter requirement in *B. napus* and contribute to the major vernalization-responsive flowering time variation in *B. napus* in a manner similar to that of *AtFLC* in *Arabidopsis* [24]. Variations at the *BnaFLC* orthologous loci that result in loss-of-function or reduced cold sensitivity alleles have been associated with different vernalization requirements and differentiation of WOR and SOR crop types in *B. napus* [25]. In addition, QTL mapping identified associations between *FRI* locus at the A3 chromosome (*BnaA3.FRI*) and vernalization response and flowering time variation in *B. napus* [18, 26, 27]. However, the molecular bases towards flowering regulation by *BnaA3.FRI* in *B. napus* are still unknown.

More recently, four *FRI* paralogues (*BnaA.FRI.a*, *BnaX.FRI.b*, *BnaX.FRI.c* and *BnaX.FRI.d*) were identified in *B. napus* [28]. In this study, we renamed the four paralogues as *BnaA3.FRI*, *BnaA10.FRI*, *BnaC3.FRI* and *BnaC9.FRI*, respectively, according to their locations in the ‘Darmor-*bzh*’ reference genome [17]. Association study further indicated that one of the *FRI* orthologues, *BnaA3.FRI*, was a major regulator of flowering time and vernalization [28]. We analyzed the sequence polymorphisms of the four *BnaFRI* paralogues in *B. napus* and performed functional analysis of *BnaA3.FRI*. By profiling the allelic variation of *BnaA3.FRI* in a panel of 174 *B. napus* accessions representing different crop types, we confirmed that the nucleotide polymorphism within this gene was associated with flowering time variation and local adaption. Expression of *BnaA3.FRI* in wild-type (WT) *Arabidopsis* Col-0 revealed that sequence variations in the coding region of *BnaA3.FRI* result in weak alleles, and led to early flowering. This study provides further molecular evidence to support predictive breeding of *B. napus* to select varieties with favorable flowering time and better adaption to latitude and seasonal shifts due to changing climate.

## Methods

### Plant materials and growth conditions

A collection of 174 *B. napus* cultivar accessions from across the world, including 17 winter-type oilseed rape (WORs), 39 spring-type oilseed rape (SORs), and 118 semi-winter-type oilseed rape (SWORs), was used for genotype analyses of *BnaA3.FRI* (Additional file 1). A subset of 30 accessions representing different crop types was used for identification of nucleotide polymorphism and haplotype determination of *BnaA3.FRI* (Additional file 2). The duration of flowering time (from the date of sowing to the date of half of the plants flowering) of all the accessions was recorded in two successive years (2013-2014) of field trials at spring growth environments (Xining, Qinghai, 36°35′ N, 101°47′ E and Lanzhou, Gansu, 36°02′ N, 103°50′ E) in North China. Seeds were sowed on May 21 and harvested on September 16, 2013 at Xining, and sowed on May 20 and harvested on September 19, 2014 at Lanzhou. At these two spring environments, plants were grown under a day length of 14 ~ 14.5 h without vernalization. All these accessions were also grown in semi-winter environment at Wuhan (30°36′ N, 104°18′ E). Seeds were sowed on October 1, 2013 and harvested on May 12, 2014. Plants were grown under a day length of 10 ~ 11 h and vernalized in the winter. The temperatures during the whole growth period of oilseed rape in the three field sites were provided in Additional file 3. Each accession was grown in the field under natural rain-fed conditions in two-row plots with 8-10 plants per row. Tissues of roots, hypocotyledonary axis, cotyledons, leaves, stems, floral buds, siliques and seeds from Tapidor (a typical WOR) were collected and used for gene expression pattern analysis. In addition, leaves before and after vernalization, floral buds and flowers from Tapidor, Ningyou7 (a typical SWOR) and Westar (a typical SOR) were collected for analysis of *BnaA3.FRI* expression. Samples from three individual plants were obtained as biological replicates.

Wild-type *A. thaliana* ecotype Columbia (Col-0) (*fri + FLC*) was used for transformation of *BnaA3.FRI*. Seeds of T1 and T2 (includes both homo- and heterozygous) transgenic lines were screened on half-strength Murashige and Skoog (MS) medium containing 50 mg/L kanamycin under a 16-h-light/8-h-dark photoperiod in greenhouse. All plants were grown under the condition of 16-h-light/8-h-dark photoperiod and 70% humidity at 23 °C. Five plants per line were selected (days to flowering was close to the mean of days to flowering of the respective line) as biological repeats for gene expression analysis.

### Nucleotide polymorphism and haplotype analysis of *BnaFRIs*

**Fig. 1:**
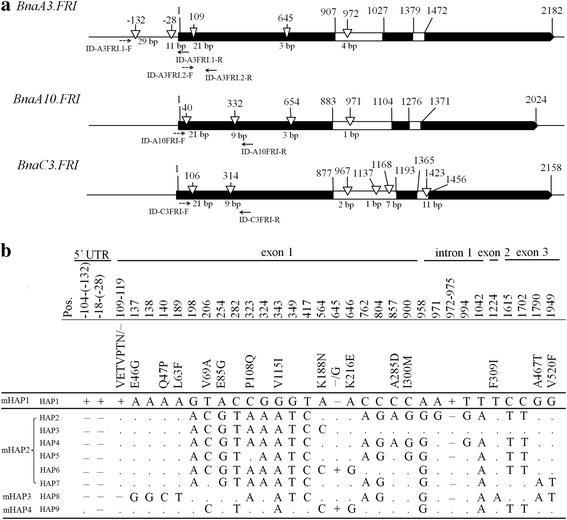
Sequence variations within *BnaFRIs*. **a** Exon-intron structure of *BnaA3.FRI*, *BnaA10.FRI*, and *BnaC3.FRI*. Black rectangles indicate exons, white rectangles indicate introns. Numbers above gene structures indicate the base pair positions that relative to the ATG start codon. The positions of these three *BnaFRIs* referred to the respective paralogue in winter-type cv. Tapidor. Triangles indicate INDELs, and the numbers below each triangle indicate the length of INDELs. Arrows mark the position and orientation of INDEL markers. **b** The nine haplotypes (HAP1-HAP9) of *BnaA3.FRI* in 30 *B. napus* accessions. The sequenced fragment included 200 bp 5’ UTR and 2.2 Kb gene regions of *BnaA3.FRI*. Base pair position of + 1 is given relative to the ‘ATG’ start codon. Pos., the positions of nucleotide and amino acid refer to the HAP1, which represented by winter-type cv. Tapidor. ‘mHAP’, marker-based haplotype. ‘HAP’, haplotype inferred by gene sequence. ‘+’ indicate insertion, ‘—’ indicate deletion. ‘.’ indicates the same sequence with HAP1

Genomic DNA of all the *B. napus* accessions was extracted from young leaves using a CTAB method [29]. The genomic fragments including ~ 0.2 kb 5’ UTR region and ~ 2.2 kb from ATG to 3’ UTR region of the *BnaA3.FRI*, and ~ 2.2 kb fragment from ATG to 3’UTR region of *BnaA10.FRI*, *BnaC3.FRI*, and *BnaC9.FRI* from five WORs (Darmor, Express, Sollox, Tapidor, Bakow) and five SORs (Drakkar, Grouse, Westar, Altex, No.2127) cultivars were amplified and sequenced. For marker-based haplotype (mHAP) analysis, four INDEL markers (ID-A3FRI.1, ID-A3FRI.2, ID-A10FRI and ID-C3FRI) were developed to determine haplotypes for *BnaA3.FRI, BnaA10.FRI* and *BnaC3.FRI* in *B. napus*, respectively (Fig. 1a, Additional file 4). Marker ID-A3FRI.1 was designed to detect the 29 bp and 11 bp INDELs in the 5’ UTR of *BnaA3.FRI*, while ID-A3FRI.2 was designed to detect the 21 bp INDELs in exon1 of *BnaA3.FRI* (Fig. 1a). ID-A10FRI and ID-C3FRI discriminated the 21 bp and 9 bp INDELs in exon1 of *BnaA10.FRI* and *BnaC3.FRI*, respectively.

The 2.4 kb fragment of *BnaA3.FRI* described above was amplified from another 20 *B. napus* accessions including SOR, SWOR, and WOR accessions, and sequenced for polymorphism analysis (Additional file 2). Sequence analysis was conducted by SeqMan Pro (DNAstar, Madison, WI, USA). The amino acid sequences of *BnaFRI* were predicted using the online software FGENESH+ (http://linux1.softberry.com/berry.phtml).

### Plasmid construction

For the functional complementation assay, a 3.8 kb genomic DNA fragment of *BnaA3.FRI* including 1.5 kb upstream sequence, the complete coding sequence, and 100 bp downstream region was amplified from each of Tapidor (a typical WOR with HAP1) and Ningyou7 (a typical SWOR with HAP2), with primers BnaA3.FRI-PF-BamHI and BnaA3.FRI-GR-SmaI (Additional file 4) using KOD-PLUS DNA Polymerase (TOYOBO, OSAKA JAPAN) and cloned into the BamHI-SmaI sites of pCAMBIA2301 to construct plasmids pBnaA3.FRI::BnaA3.FRI (pHAP1::HAP1 and pHAP2::HAP2).

In order to investigate the effects of different *BnaA3.FRI* alleles, promoter-swap constructs were generated. The promoters (a DNA fragment of 1.5 kb upstream of the *BnaA3.FRI*) were amplified from HAP1 (Tapidor) and HAP2 (Ningyou7) using primers BnaA3.FRI-PF-BamHI and BnaA3.FRI-PR-SmaI, and cloned into the BamHI-SmaI sites of pCAMBIA2301 to construct intermediate vectors. The coding sequences of *BnaA3.FRI* from four different haplotypes were amplified from Tapidor (HAP1), Ningyou7 (HAP2), 3B014-2 (HAP3), and Altex (HAP8), respectively, using primers BnaA3.FRI-GF-SmaI and BnaA3.FRI-GR-EcoRI, and introduced into the SmaI-EcoRI sites of the intermediate vectors to generate plasmids pHAP1::HAP2, pHAP1::HAP3, pHAP1::HAP8 and pHAP2::HAP1. In plasmid pHAP2::HAP1, the coding sequence of HAP1 was driven by the promoter from HAP2. In plasmids pHAP1::HAP2, pHAP1::HAP3, and pHAP1::HAP8, the coding regions of HAP2, 3, and 8 were driven by the promoter from HAP1. All primers used in this study are listed in Additional file 4.

To analyze the expression pattern of *BnaA3.FRI*, a 1.4 kb promoter fragment from Tapidor (HAP1) was amplified using primers proF-BamHI and proR-HindIII and then cloned into the BamHI-HindIII sites of pCAMBIA2301-GUS, which was generated from pCAMBIA2301 by inserting the *β-glucuronidase* (*GUS*) gene into the HindIII–PmlI sites, to construct the pBnaA3.FRI::GUS plasmid. For cellular localization analysis*,* full-length of *BnaA3.FRI* cDNA sequence (1729 bp) was isolated from Tapidor (HAP1) using primers BnaA3.FRI–F-PmlI and BnaA3.FRI-R-AsiSI (Additional file 4) and inserted into the PmlI-AsiSI sites of pDOE20 [30] to generate mVenus-BnaA3.FRI (YFP-BnaA3.FRI) fusion protein. Meanwhile, a nuclear marker Ghd7 [31] was introduced into the BamHI-XbaI sites of pDOE20 to generate the mTurquoise2-GHD7 (CFP-GHD7) fusion protein. In this way, two fusion proteins co-express in a single construct.

### Plant transformation and phenotyping

For expression pattern determination and functional analysis of *BnaA3.FRI*, *Agrobacterium tumefaciens* strain GV3101 harboring plasmid pHAP1::HAP1, pHAP1::HAP2, pHAP1::HAP3, pHAP1::HAP8, pHAP2::HAP2, pHAP2::HAP1, or pBnaA3.FRI::GUS was used to transform *A. thaliana* accession Col-0 by a floral dip method [32]. T1 and T2 transgenic plants were screened on half-strength MS plates containing 50 mg/L kanamycin. Flowering time of 15 T2 transgenic plants of each line was recorded. Flowering time was determined by the mean rosette leaf number or the mean days from sowing to bolting time, with the bolting time determined by when the inflorescence stem was 1 cm high.

### RT-PCR analysis

Total RNA was isolated using an RNAprep Pure Plant kit (BioTeKe, China) according to the manufacturer’s instructions. The concentration of RNA was determined by spectrometry (NanoDrop; Thermo Scientific, USA). RNase-free DNaseI (Thermo Scientific, USA) was used to remove contaminated DNA, and then a RevertAid First Strand cDNA Synthesis Kit (Thermo Scientific, USA) was used to reverse transcribe RNA samples. Quantitative RT-PCR (qRT-PCR) was performed to analyze the expression levels of *BnaA3.FRI* in *B. napus*, and *BnaA3.FRI* and *AtFLC* in transgenic plants. Amplification of cDNA was conducted using a SYBR Green master mixture (BioRad, USA) with a LightCycler 96 (Roche, USA). The expression level of Epsin N-terminal homology (*BnaENTH*) [33] and *ACTIN2* gene (AT3G18780) was used as an internal control in *B. napus* and *A. thaliana*, respectively. The sequence of primers used for quantitative RT-PCR analyses is listed in Additional file 4.

### Histochemical staining and subcellular localization

Tissues including seedlings, leaves, stems, floral buds, and siliques from the pBnaA3.FRI::GUS T1 and T2 transgenic lines were used for GUS staining to determine the expression pattern of *BnaA3.FRI*. Tissues were incubated in the staining solution containing 1 g/L X-gluc (Sigma-Aldrich, USA) at 37 °C overnight followed by decolorization with 70% ethanol [34]. A transient expression experiment was performed in tobacco (*Nicotiana benthamiana* cv. SR1) leaves to analyze the subcellular localization of *BnaA3.FRI* according to Voinnet et al. [35]. The *Agrobacterium* GV3101 cells containing the pDOE20 recombinant plasmid that expresses CFP-GHD7 and YFP-BnaA3.FRI, also expresses the silencing suppressor p19 of *Tomato bushy stunt virus*, were harvested and re-suspended in the solution of 10 mM MES-KOH, pH 5.6, containing 10 mM MgCl_2_ and 150 mM acetosyringone to a final density of 0.8 at 600 nm (OD_600_). The *Agrobacterium* suspension was injected into expanded leaves of 6-week-old tobacco plants. Three days after injection, the leaves were observed with a laser scanning confocal imaging system (TCS SP2, Leica, Germany).

### Statistical analysis

For flowering time and gene expression comparison between each two data sets, we performed *F* test to compare the significant level of sample variances (α = 0.05), and then equal variance or unequal variance two-tailed *t*-test was conducted according to the result of *F* test (equal variance *t*-test when the *P* value of *F* test equal to or above 0.05, unequal variance t-test when the *P* value of *F* test below 0.05).

## Results

### Sequence variations of *BnaFRI* paralogues in *B. napus*

To investigate sequence variations of the four *BnaFRIs* (*BnaA3.FRI*, *BnaA10.FRI*, *BnaC3.FRI* and *BnaC9.FRI*) in different crop types of *B. napus*, the 2.4 kb genomic fragment of *BnaA3.FRI* (including 200 bp 5’ UTR and ~ 2.2 kb gene region), and the ~ 2.2 kb genomic fragments of *BnaA10.FRI*, *BnaC3.FRI* and *BnaC9.FRI* (from ATG to ~ 50 bp 3’ UTR) were amplified from five SORs and five WORs and sequenced. Four INDELs and 26 single nucleotide polymorphisms (SNPs) were identified in *BnaA3.FRI* (Additional file 5). Of the four INDELs, two (29 bp and 11 bp) were located in the 5’ UTR, one (21 bp) in exon 1, and the other (4 bp) in intron 1 (Fig. 1a). Of the 26 SNPs, 14 result in amino acid substitutions (Fig. 1b). In *BnaA10.FRI*, a total of four INDELs and 26 SNPs were identified (Additional file 5). Three (21 bp, 9 bp and 3 bp) of the INDELs located in exon 1, and the other (1 bp) in intron 1 (Fig. 1a). Eleven of the SNPs result in amino acid substitutions. Six INDELs and 44 SNPs were identified in *BnaC3.FRI* (Fig. 1a, Additional file 5). Of the six INDELs, two (21 bp and 9 bp) were located in exon 1, three (2 bp, 1 bp and 7 bp) in intron 1, and the other (11 bp) in intron 2 (Fig. 1a). Fourteen of the SNPs cause amino acid substitutions. All the INDELs in exons of the three paralogues result in in-frame amino acid insertion/deletion. Although such variations were found in these three *BnaFRI* paralogues, no such sequence variation was detected in *BnaC9.FRI*.

To explore the haplotype variations of *BnaFRIs* in different *B. napus* crop types, a panel of 174 *B. napus* accessions, including 39 SORs, 118 SWORs, and 17 WORs (Additional file 1) were genotyped using the four INDEL markers, ID-A3FRI.1, ID-A3FRI.2, ID-A10FRI, and ID-C3FRI (Fig. 1a). Haplotypes detected by these INDEL markers are named as marker-based haplotypes (mHAPs) (Fig. 1b). In total, four *BnaA3.FRI* mHAPs (mHAP1, 2, 3, and 4), three *BnaA10.FRI* mHAPs (mHAP1, 2, and 3), and four *BnaC3.FRI* mHAPs (mHAP1, 2, 3, and 4) were identified in the 174 accessions (Table 1). For *BnaA3.FRI*, 40 accessions were inferred as mHAP1 (22.99%), 106 as mHAP2 (60.92%), 25 as mHAP3 (14.37%), and 3 as mHAP4 (1.7%). For *BnaA10.FRI*, 89 accessions were inferred as mHAP1 (51.14%), 37 as mHAP2 (21.26%), and 48 as mHAP3 (27.59%). For *BnaC3.FRI*, 125 accessions were inferred as mHAP1 (71.84%), 33 as mHAP2 (18.97%), 15 as mHAP3 (8.62%), and 1 as mHAP4 (0.57%) (Table 1).

**Table 1:** Haplotype of *BnaFRIs* in a panel of 174 *B. napus* by INDEL markers

	Fequency	INDELs		
*BnaA3.FRI*		Promoter^*a*^ (29 bp)	Promoter (11 bp)	Exon1 (21 bp)
mHAP1^*b*^	40 (22.99%)	+^*c*^	+	+
mHAP2	106 (60.92%)	–	–	+
mHAP3	25 (14.37%)	–	–	–
mHAP4	3 (1.7%)	+	–	+
*BnaA10.FRI*		Exon1 (21 bp)	Exon1 (9 bp)	
mHAP1	89 (51.14%)	+	+	
mHAP2	37 (21.26%)	–	–	
mHAP3	48 (27.59%)	+	–	
*BnaC3.FRI*		Exon1 (21 bp)	Exon1 (9 bp)	
mHAP1	125 (71.84%)	+	+	
mHAP2	33 (18.97%)	–	–	
mHAP3	15 (8.62%)	–	+	
mHAP4	1 (0.57%)	+	–	

^a^the location and length of INDEL

^b^Marker-based haplotype. For each paralogue, haplotype in winter oilseed rape cv. Tapidor was inferred as mHAP1

^c^+, insertion; −, deletion

To further explore sequence variations of *BnaA3.FRI*, the ~ 2.4 kb genomic fragment of *BnaA3.FRI* mentioned above was amplified from another 20 *B. napus* accessions representing different crop types, and sequenced (Additional file 2). In addition to the previously identified 30 polymorphic sites (4 INDELs and 26 SNPs) in the 10 *B. napus* accessions, three more polymorphic sites were identified in exon 1, including one 3-bp INDEL and two SNPs (Fig. 1b). All sequence variations detected across the 30 sequenced accessions could be classified into nine haplotypes (HAP1-HAP9) (Fig. 1b). HAP1 was identified in 10 accessions, HAP2 in eight accessions and HAP8 in five accessions (Additional file 2). All the WOR carried HAP1, which corresponded to the mHAP1 inferred by INDEL markers. HAP2 to HAP7 corresponded to mHAP2, HAP8 to mHAP3, and HAP9 corresponded to mHAP4 (Fig. 1b).

### Associations of *BnaFRIs* with crop type and flowering time of *B. napus*

To check associations between each *BnaFRI* paralogue and *B. napus* crop type, Chi-square analyses were performed to test the distributions of *BnaFRI* mHAPs within each crop type. For *BnaA3.FRI*, all the WOR accessions only contained mHAP1, the majority of SWOR accessions contained mHAP2 (97/118, 82.2%), while the SOR accessions contained three major haplotypes, mHAP1 (15/39, 38.46%), mHAP2 (9/39, 23.08%) and mHAP3 (14/39, 35.90%), and a minor haplotype mHAP4 (1/39, 2.56%). Chi-square analysis indicated that all the crop types had a biased number of lines with each of the *BnaA3.FRI* mHAPs (Table 2), which denied the null hypothesis (*H*_*0*_) that *BnaA3.FRI* mHAPs follow the same distribution among crop types. For *BnaA10.FRI*, the frequency of mHAP1, mHAP2, and mHAP3 in SOR accessions were 15.58% (6/39), 51.28% (20/39), and 33.33% (13/39), respectively, and 58.47% (69/118), 11.86% (14/118), and 29.66% (35/118), respectively, in SWOR. The WOR accessions only contained mHAP1 and mHAP2, which the frequencies were 82.35% (14/17) and 17.65% (3/17), respectively. Chi-square analysis indicated that *BnaA10.FRI* mHAPs also displayed a biased distribution among the three crop types. For *BnaC3.FRI*, the mHAP1 was the most represented mHAP in the SOR and SWOR accessions (33/39, 84.62%, and 87/118, 73.73%, respectively), and the mHAP2 was the most represented mHAP in WOR accessions (70.59%). Chi-square analysis showed that only WOR had a biased distribution of *BnaC3.FRI* mHAPs (Table 2). These results indicated that all the three *BnaFRI* paralogues may involve in the crop type differentiation in *B. napus*.

**Table 2:** Chi-square test for the *BnaFRIs* haplotype distributions within different crop type of *B. napus*

Chi-square test for *BnaA3.FRI* haplotypes and crop types						
	mHAP1^*a*^	mHAP2	mHAP3	mHAP4	Total	*χ2* (3^*b*^)
Spring	15	9	14	1	39	25.97^***c^
Semi-winter	8	97	11	2	118	24.35^***^
Winter	17	0	0	0	17	56.95^***^
Chi-square test for *BnaA10.FRI* haplotypes and crop types						
	mHAP1	mHAP2	mHAP3	Total		*χ2* (2)
Spring	6	20	13	39		26.75^***^
Semi-winter	69	14	35	118		6.33^*^
Winter	14	3	0	17		8.03^*^
Chi-square test for *BnaC3.FRI* haplotypes and crop types						
	mHAP1	mHAP2	mHAP3	mHAP4	Total	*χ2* (3)
Spring	33	2	4	0	39	5.26
Semi-winter	87	19	11	1	118	0.83
Winter	5	12	0	0	17	29.63^***^

*H*_*0*_: *BnaFRIs* mHAPs follow the same distribution among the three *B. napus* crop types

^a^mHAP, haplotype based on INDEL markers

^b^degrees of freedom

^c^*** *p* < 0.001, * *p* < 0.05

**Fig. 2:**
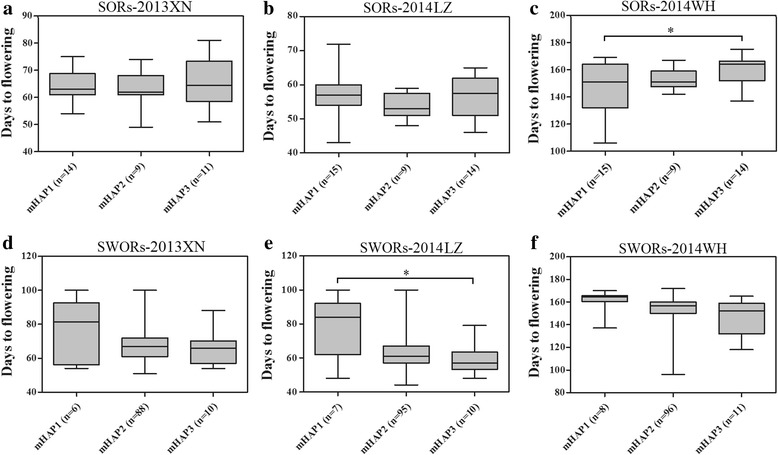
The effect of different *BnaA3.FRI* haplotypes on mean days to flowering in SORs (**a**-**c**) and SWORs (**d**-**f**) in the three growing conditions (2013XN = at year 2013, Qinghai, spring environment; 2014LZ = at year 2014, Gansu, spring environment; 2014WH = at year 2014, Wuhan, semi-winter environment)**.** Numbers in brackets indicate the number of accessions of each mHAP. ‘mHAP’ marker-based haplotype. *, significant difference according to *t*-test (*α* = 0.05), *P* < 0.05

To dissect the associations of *BnaFRIs* and flowering time, the flowering time of different *BnaFRI* mHAPs within each crop type were compared using *t*-tests. For *BnaA3.FRI*, the means of flowering date of SOR accessions with mHAP1, mHAP2, and mHAP3 had no difference in spring environments at 2013XN and 2014LZ (Fig. 2a, b). However, accessions with mHAP1 and mHAP3 displayed a significant difference in flowering time when grown in the semi-winter environment of 2014WH (146.2 ± 17.18 d vs. 160.1 ± 9.7 d, *P* = 0.0218) (Fig. 2c). In SWOR, accessions with mHAP1 flowered later than those with mHAP3 in all environments (Fig. 2d, e, and f), and displayed a significant difference in flowering time when grown in the spring environment of 2014LZ (78.1 ± 16.9 d vs. 59.1 ± 8.5 d, *P* = 0.0113) (Fig. 2e). The SWOR accessions with mHAP2 showed an intermediate flowering time between mHAP1 and mHAP3 (Fig. 2d, e, and f). The WOR accessions, which only contained mHAP1, flowered extremely late or did not flower when grown in the two spring environments of 2013XN and 2014LZ, and flowered (178.5 ± 5.08 d) much later than all SOR and SWOR accessions when grown in the winter environment of 2014WH (Additional file 1). Different from *BnaA3.FRI*, the accessions with different *BnaA10.FRI* or *BnaC3.FRI* mHAPs within each crop type did not display significant variations on flowering time in all assessed environments (Additional files 6, 7). For the combinations of the three *BnaFRI* haplotypes, the significant difference on flowering time was only observed between the SOR accessions with *BnaA3.FRI* mHAP1 + *BnaC3.FRI* mHAP1 + *BnaA10.FRI* mHAP2 (133.4 ± 15.89 d) and accessions with *BnaA3.FRI* mHAP3 + *BnaC3.FRI* mHAP1 + *BnaA10.FRI* mHAP2 (164.5 ± 2.36 d) in 2014WH environment (Additional file 8), which was mainly caused by the mHAP difference of *BnaA3.FRI*. These results suggested that, among the three *BnaFRIs*, only *BnaA3.FRI* is associated with flowering time variation in *B. napus*. We thus focused our study on functional analysis of *BnaA3.FRI*.

### Expression and subcellular localization of *BnaA3.FRI*

**Fig. 3:**
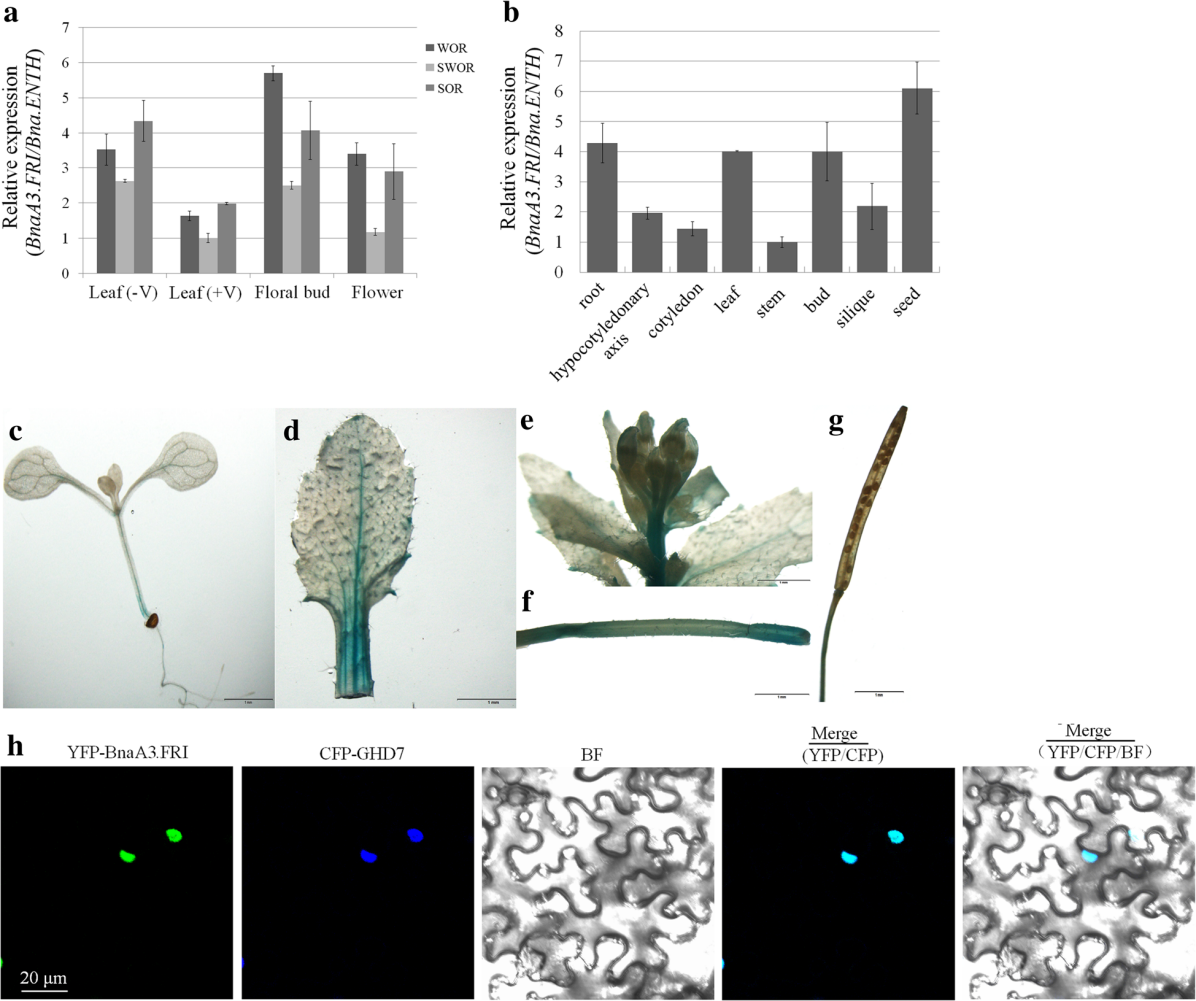
Expression and subcellular localization analysis of *BnaA3.FRI*. **a** Expression of *BnaA3.FRI* between different crop types of *B. napus*. ‘+V’ indicates tissues after vernalization, ‘-V’, indicates tissues before vernalization. **b** qRT-PCR analysis of *BnaA3.FRI* gene expression in various tissues, (**c**-**g**) *BnaA3.FRI* promoter-driven GUS expression, seedling (**c**), rosette leaf (**d**), flowers (**e**), stem (**f**), and silique (**g**). Bars = 1 mm. **h** Subcellular localization of *BnaA3.FRI*. Co-expression of YFP-BnaA3.FRI and CFP-GHD7 fusion proteins in leaf epidermal cells of tobacco. From left to right: YFP fluorescence, CFP fluorescence showing the nuclei, bright-field (BF), merged image of YFP/CFP, and merged image of YFP/CFP/BF. Bar = 20 μm

The expression level of *BnaA3.FRI* was analyzed in leaves before and after vernalization, floral buds, and flowers of the three crop types represented by Tapidor (WOR with HAP1), Ningyou7 (SWOR with HAP2), and Westar (SOR with HAP2) using quantitative RT-PCR (qRT-PCR). As shown in Fig. 3a, *BnaA3.FRI* expressed in all the four tissues, and displayed a lowest level in leaves after vernalization and highest in floral buds.

To explore the expression pattern of *BnaA3.FRI*, we checked the mRNA transcripts of *BnaA3.FRI* in roots, hypocotyledonary axes, cotyledons, leaves, stems, floral buds, siliques and seeds from the WOR variety Tapidor (Fig. 3b). *BnaA3.FRI* transcribed in all tissues but showed much higher expression level in roots, leaves, floral buds and seeds (Fig. 3b). Transgenic *Arabidopsis* lines harboring the construct pBnaA3.FRI::GUS, which contained the 1.5 kb promoter region of *BnaA3.FRI* from Tapidor (HAP1), were used for GUS staining. Histochemical staining revealed strong GUS activity in roots, leaves, and flowers (Fig. 3c–g).

Previous studies in *Arabidopsis* demonstrated that FRI acts as a scaffold protein to recruit several chromatin modifiers in nucleus and epigenetically modify the key flowering regulator *FLC* [11, 13]. *BnaA3.FRI* is predicted to translate a putative protein containing 596 amino acids, which carries the conserved ‘Frigida’ domain [36], and shares 58% identity in amino acid sequence with AtFRI (AF228500) [15, 28]. To explore the subcellular localization of the *BnaA3.FRI* protein, the plasmid pDOE20, that co-expresses fusion proteins YFP-BnaA3.FRI and CFP-Ghd7, was introduced into tobacco leaves for transient expression analysis. The results demonstrated that *BnaA3.FRI* was co-localized with Ghd7 in the nucleus (Fig. 3h).

### Functional analysis of *BnaA3.FRI*

**Fig. 4:**
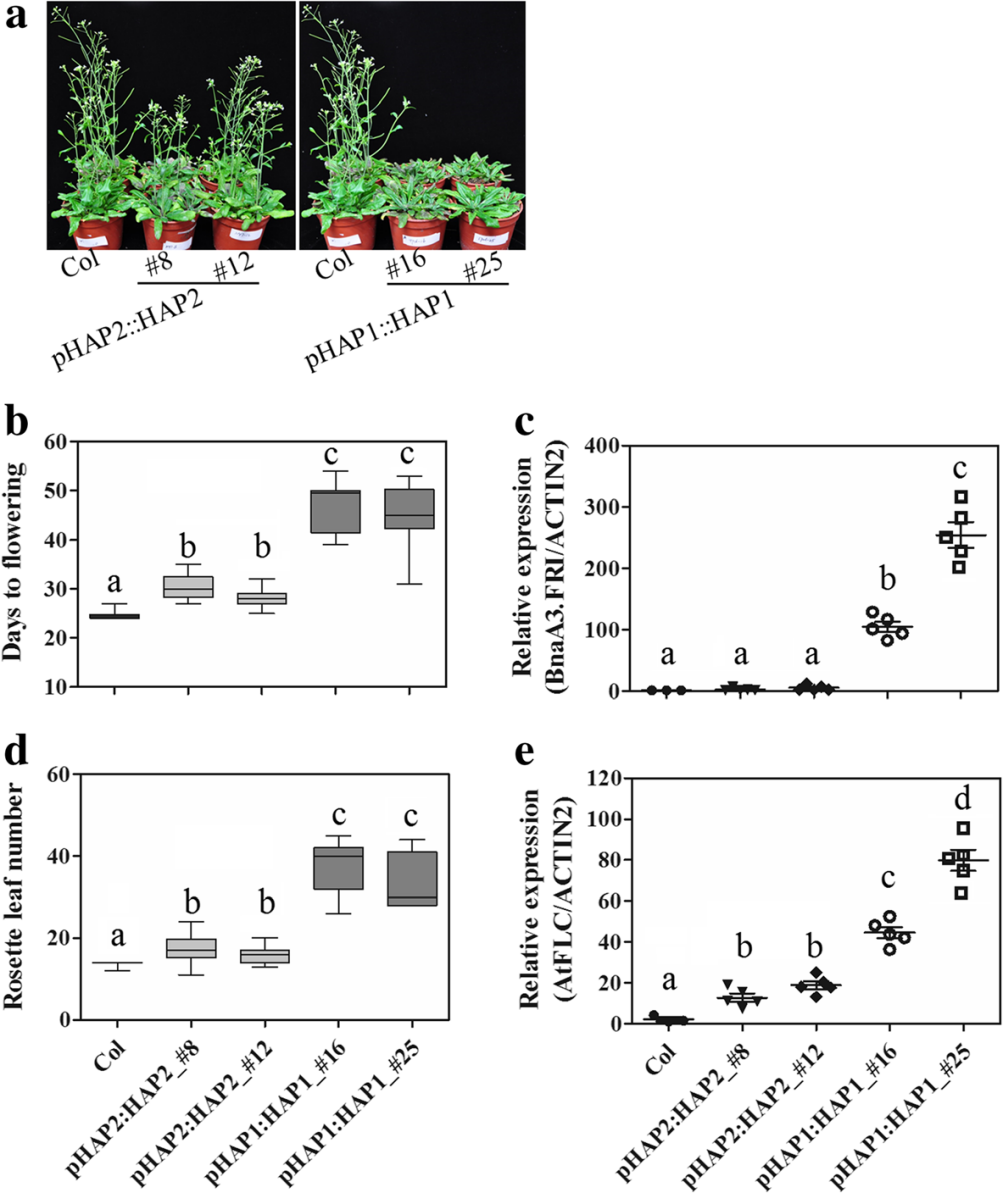
Functional analysis of *BnaA3.FRI* in *Arabidopsis*. Variance of phenotype for flowering time and gene expression levels between transgenic lines harboring different *BnaA3.FRI* haplotypes and Col-0. **a** The phenotype at flowering stage, (**b** and **d**) days to flowering and rosette leaf numbers at bolting stage, (**c** and **e**) relative expression level of *BnaA3.FRI* and *AtFLC* in five plants for each transgenic line and 3 plants for WT Col-0. Letters indicate significant differences according to *t*-test (*α* = 0.05)

In *Arabidopsis*, *FRI* is the major determinate of flowering time variation [15]. In order to understand the effect of *BnaA3.FRI* variation on flowering time, binary vectors pBnaA3.FRI::BnaA3.FRI that contained a 3.8 kb DNA fragment including 1.5 kb 5’ UTR, 2.2 kb coding and 100 bp downstream regions of *BnaA3.FRI* HAP1 (pHAP1::HAP1) or HAP2 (pHAP2::HAP2) were transformed into *A. thaliana* Columbia (Col-0) (*fri + FLC*), respectively. Two independent T2 transgenic lines each were selected from the HAP1 and HAP2 positive transgenic plants. One-way ANOVA and *t*-test indicated that the two T2 transgenic lines harboring *BnaA3.FRI* HAP1 (47.2 ± 3.79 d and 37.4 ± 6.43 l; 46.71 ± 3.15 d and 33.3 ± 6.25 l) or HAP2 (30.22 ± 2.74 d, and 17.6 ± 3.35 l; 28.11 ± 1.29 d and 16 ± 1.91 l) flowered significantly later than WT Col-0 (24.17 ± 0.37 d and 13.75 ± 0.66 l) (*P* < 0.01) (Fig. 4b, d). Moreover, both days to flowering and rosette leaf number of the two HAP1 transgenic lines were significantly greater than those of the two HAP2 transgenic lines (Fig. 4a–d), suggesting that HAP1 is a strong allele.

To confirm whether the later flowering of transgenic lines was caused by elevated *AtFLC* expression level due to the introduction of a functional *BnaA3.FRI* gene, qRT-PCR was performed to compare the expression levels of *BnaA3.FRI* and *AtFLC* between transgenic lines and WT Col-0. In accordance with the flowering time variations between these lines, the *BnaA3.FRI* and *AtFLC* levels in the two HAP2 transgenic lines were significantly lower than those in the two HAP1 transgenic lines (Fig. 4c and e).

### Effects of sequence variations in promoter and coding regions of *BnaA3.FRI*

**Fig. 5:**
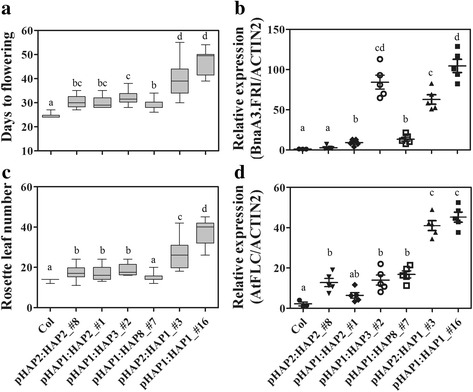
The effects of polymorphic sites of *BnaA3.FRI*. Variance of phenotype for flowering time and gene expression levels between transgenic lines harboring different *BnaA3.FRI* haplotypes and Col-0. **a** and **c** days to flowering and rosette leaf numbers at bolting stage, (**b** and **d**) relative expression level of *BnaA3.FRI* and *AtFLC* in five plants for each transgenic line and 3 plants for WT Col-0. Letters indicate significant differences according to *t*-test (*α* = 0.05)

To validate whether variations in promoter resulted in differential expression of *BnaA3.FRI*, or whether the variations in coding sequence cause functional defects of the *BnaA3.FRI* protein, combinations of promoter and coding sequence of different haplotypes (pHAP1::HAP2, pHAP1::HAP3, pHAP1::HAP8, and pHAP2::HAP1) were transformed into Col-0 for phenotypic and transcriptional analysis*.* All transgenic lines harboring recombinant *BnaA3.FRI* haplotypes showed later flowering when compared to WT Col-0 (Fig. 5a, Additional file 9). The transgenic lines that harbored pHAP2::HAP1 (41.5 ± 7.29 d) flowered significantly later than lines harboring pHAP1::HAP2 (30.91 ± 3.94 d), pHAP1::HAP3 (32.1 ± 2.72 d), or pHAP1::HAP8 (35 ± 3.13 d) (*P* < 0.01) (Fig. 5a). Moreover, the rosette leaf number for pHAP2::HAP1 (26.9 ± 7.75 leaves) was also significantly higher than for the transgenic lines pHAP1::HAP2 (16.9 ± 3.26 leaves) or pHAP1::HAP3 (18.75 ± 2.65 leaves) (*P* < 0.01) (Fig. 5c). However, no significant difference in days to flowering was observed between the transgenic lines of pHAP2::HAP1 and pHAP1::HAP1, or between pHAP1::HAP2 and pHAP2::HAP2 (Fig. 5a). These results indicate that variations in the coding sequence of *BnaA3.FRI* leading to weak alleles, and causing variations in flowering time.

The *BnaA3.FRI* expression levels in pHAP1::HAP3, pHAP2::HAP1, and pHAP1::HAP1 transgenic lines were significantly higher than the transgenic lines harboring pHAP2::HAP2, pHAP1::HAP2, and pHAP1::HAP8, and WT Col-0 (*P* < 0.01) (Fig. 5b). While driven by the same promoter, the *BnaA3.FRI* mRNA levels showed significant differences among the transgenic lines of pHAP1::HAP1, pHAP1::HAP2, pHAP1::HAP3, and pHAP1::HAP8, and also between pHAP2::HAP1 and pHAP2::HAP2 (Fig. 5b), suggesting that there might have an important *cis*-element necessary for transcription within the coding region. The transcription levels of *AtFLC* were up-regulated in all transgenic lines when compared to WT Col-0 (Fig. 5d). It is worth noting that the expression level of *BnaA3.FRI* did not correlate with the mRNA level of *AtFLC* (Fig. 5b, d). However, the expression level of *AtFLC* was proportional to the flowering time of these transgenic lines (Fig. 5a, d). Taken together, our results demonstrate that HAP1 is stronger than other haplotypes and mutations in the coding sequence of *BnaA3.FRI* result in weak alleles, which contribute to variation in flowering time.

## Discussion

### Potential functional divergence of different *FRI* orthologues in *B. napus*

Four *FRI* orthologues, *BnaA3.FRI*, *BnaA10.FRI*, *BnaC3.FRI* and *BnaC9.FRI*, had previously been identified in *B. napus* [28, 37]. In this study, a large number of sequence variations including INDELs and SNPs were identified within the coding sequences of *BnaA3.FRI*, *BnaA10.FRI* and *BnaC3.FRI* (Additional file 5)*.* In contrast to the high level of polymorphism observed in the other *B. napus FRI* genes, no sequence variation was identified in *BnaC9*.*FRI*. Correspondingly, the orthologue of *BnaC9*.*FRI* in *B. oleracea*, *BolC*.*FRI.b*, was also highly conserved in different crop types of *B. oleracea* [38]. Moreover, *BnaC9.FRI* in *B. napus* displayed a high level of mRNA transcripts in both vernalized and non-vernalized tissues [38]. These results suggest *BnaC9.FRI* may be functionally important, but requires further investigation.

Marker-based haplotype analysis of *BnaFRIs* in a panel of 174 *B. napus* identified four, three, and four mHAPs for *BnaA3.FRI*, *BnaA10.FRI* and *BnaC3.FRI*, respectively. Chi-square analyses showed that the WOR accessions had a biased distribution in mHAPs of all the three *BnaFRIs*, and the SOR and SWOR had a biased distribution in mHAPs of *BnaA3.FRI* and *BnaA10.FRI* (Table 2). However, of the three *BnaFRIs,* only *BnaA3.FRI* variation was associated with flowering time variation in *B. napus* (Fig. 2), and no association was identified between flowering time and combinations of *BnaFRIs* alleles (Additional file 8). In accordance with previous studies, our results suggested that *BnaA3.FRI* plays important roles in vernalization response, crop type differentiation and flowering time variation in *B. napus* [18, 26–28]. However, associations of *BnaC3.FRI* and *BnaA10.FRI* with vernalization response and flowering time have not documented. Sequence analyses indicated that the two *BnaC3.FRI* haplotypes shared extremely high amino acid identity to the two *BolC.FRI.a* alleles (99.3% between *BnaC3.FRI* -mHAP1 and *BolC.FRI.a*-E8, and 99.8% between *BnaC3.FRI* -mHAP2 and *BolC.FRI.a*-E1) (Additional file 10), the corresponding orthologues in *B. oleracea* [38]. Moreover, *BnaC3.FRI* -mHAP1 and *BolC.FRI.a*-E1 were over-represented in both winter type *B. napus* and *B. oleracea* [38]. The two *BolC.FRI.a* alleles have been demonstrated to equally delay flowering time in *Arabidopsis* [38], indicating that sequence variations in *BolC.FRI.a* do not affect its function, which is in accordance with our result that *BnaFRI.C3* did not associate with flowering time variation in *B. napus* (Additional file 7). Thus, further study will need to speculate the specific functions of *BanC3.FRI* and *BnaA10.FRI*.

### Variation of *BnaA3.FRI* is tightly associated with flowering time variation in *B. napus*

Previous linkage and association studies indicated that *BnaA3.FRI* is an important flowering regulator [28] in *B. napus*. Extensive sequence variations were identified in both 5’ UTR and coding region of *BnaA3.FRI* (Fig. 1), and could be classified into four mHAPs across the 174 acceessions. The distribution of haplotypes showed strong association with crop type differentiation, as all the WOR accessions contained mHAP1, the majority of SWOR contained mHAP2, while the SOR did not have a major haplotype (Table2). Expression of different *BnaA3.FRI* haplotypes in *Arabidopsis* showed that the function of mHAP1 (corresponding to HAP1) is much stronger than mHAP2 (corresponding to HAP2) and mHAP3 (corresponding to HAP8) (Figs. 4 and 5). Thus, our results establish that the *BnaA3.FRI* mHAP1 is the wild type allele, and confers the vernalization requirement and late flowering of WOR.

Further investigation of the relationship between *BnaA3.FRI* haplotypes and variations in flowering time in both SWOR and SOR indicated that different *BnaA3.FRI* haplotypes were consistent with significant differences in flowering time (Fig. 2). As expected, the SWOR with mHAP1 flowered later than those with mHAP2 or mHAP3, especially in spring environment (Fig. 2e). The later flowering of SWOR with mHAP1 may result from the activated expression of *BnaFLCs*. In *Arabidopsis*, *FRI* is a major determinant of vernalization response and flowering time variation, which is primarily achieved by activating the expression of *FLC*, and so loss-of-function mutation of *FRI* could lead to early flowering [15, 39]. The vernalization pathway appears to be conserved in *B. napus,* since QTLs corresponding to *BnaA3.FRI* and *BnaA10.FLC* explained the majority of vernalization response and flowering time variations in different populations [18, 40]. However, it was interesting to note that the SOR containing mHAP1 flowered earlier than those with mHAP2 or mHAP3 (Fig. 2c). This may because the major *FLC* locus (*BnaA10.FLC*) in SORs, which is a key gene in vernalization pathway and acts down-stream of *FRI*, is mutated (our unpublished data). On the other hand, a similar result has also been documented in *Arabidopsis*, where summer-annual accessions with functional *FRI* alleles accelerate flowering relative to those with nonfunctional *FRI* alleles [16]. Both the SOR and summer-annual *Arabidopsis* have an early flowering growth type and can initiate flowering without vernalization via the photoperiod pathway. Thus, further investigation will be needed to identify the specific flowering time control mechanism of *BnaA3.FRI* in SOR, and whether it works independent from *FLC* or vernalization.

### Variation in coding region of *BnaA3.FRI* results in weak alleles

In this study, we identified 33 polymorphic sites within the 5’ UTR and coding region of *BnaA3.FRI* in 30 *B. napus* accessions (Fig. 1b). An excess of non-synonymous mutations were identified within the coding region of *BnaA3.FRI*, with 14 SNPs and two INDELs predicted to cause amino acid substitution or deletion (Fig. 1b). The majority of these polymorphic sites were common between this and a previous study [28], and no frame shift or stop codons mutations were identified within *BnaA3.FRI*. This differs from the *FRI* mutation types in *Arabidopsis*, where the major two mutant alleles, *FRI* (*Col*) and *FRI* (*Ler*), are loss-of-function and vernalization insensitive [10, 15, 39, 41]. Functional analysis of four different *BnaA3.FRI* haplotypes in *Arabidopsis* (HAP1, HAP2, HAP3, and HAP8) indicated they were all functional (Fig. 5). However, HAP1 showed a much stronger function in delaying flowering time than the other three haplotypes. These results suggest that these sequence variations in the coding region of *BnaA3.FRI* result in weak alleles, and contribute to subtle flowering time variation in *B. napus* and appear to be under ongoing active selection since domestication. Candidate gene association analysis by Wang et al. [28] identified several SNPs that associate with flowering time variation. We conclude that further studies will need to identify the key amino acid sites that influence the function of BnaA3.FRI protein. The two INDELs in 5’ UTR differentiated two major kinds of promoters. However, transgenic analysis demonstrated they had an equivalent function under the conditions tested (Fig. 5). This indicates that these variations in promoter had no or little effect on the expression of *BnaA3.FRI*.

## Conclusions

In this study we identified a number of sequence variations in the coding region as well as 5’ UTR region of *BnaA3.FRI*, and investigated the effects of these variations on flowering time by association and transgenic analysis in *B. napus* and *Arabidopsis*, respectively. While major variations observed in coding sequence of *BnaA3.FRI* confer weak alleles that lead to flowering time variation, variations in promoter had no or little effect on the expression of *BnaA3.FRI*. These results thus provide useful information for selection of appropriate alleles in *B. napus* breeding for optimal flowering time.

## Additional files

Additional file 1: Accession information of the 174 *B. napus* and their *BnaA3.FRI* haplotypes. (XLSX 20 kb)

Additional file 2: Sequence variations and haplotypes of *BnaA3.FRI* in 30 *B. napus*. (XLSX 14 kb)

Additional file 3: The temperature data for the whole growth period of oilseed Rapa in the three field sites. (XLSX 20 kb)

Additional file 4: Primers used in this study. (XLSX 12 kb)

Additional file 5: Polymorphic sites within *BnaA3.FRI*, *BnaA10*.*FRI*, and *BnaC3*.*FRI*. (XLSX 14 kb)

Additional file 6: The effect of different *BnaA10.FRI* haplotypes on mean days to flowering in SORs (accession numbers of mHAP1-3 were 6, 20, and 13, respectively) (A-C) and SWORs (accession numbers of mHAP1-3 were 69, 14, and 35, respectively) (D-F). Numbers above each box indicate the means of days to flowering for the three growing conditions (2013XN = at year 2013, Qinghai, spring environment; 2014LZ = at year 2014, Gansu, spring environment; 2014WH = at year 2014, Wuhan, semi-winter environment). ‘mHAP’ marker-based haplotype. (TIFF 421 kb)

Additional file 7: The effect of different *BnaC3.FRI* haplotypes on mean days to flowering in SORs (accession numbers of mHAP1-3 were 33, 2, and 4, respectively) (A-C), and SWORs (accession numbers of mHAP1-3 were 87, 19, and 11, respectively) (D-F), and WOR (accession numbers of mHAP1 and 2 were 5 and 12, respectively) (G). Numbers above each box indicate the means of days to flowering for the three growing conditions (2013XN = at year 2013, Qinghai, spring environment; 2014LZ = at year 2014, Gansu, spring environment; 2014WH = at year 2014, Wuhan, semi-winter environment). ‘mHAP’ marker-based haplotype. (TIFF 590 kb)

Additional file 8: The effect of combinations of *BnaFRIs* haplotypes on mean days to flowering in SORs (A-C), SWORs (D-F), and WOR (G). 1 + 1 + 1 indicates the combinations of *BnaA3.FRI* + *BnaC3*.*FRI* + *BnaA10*.*FRI*, and so on for the rest. Numbers in brackets indicate the accession number of each combination. Numbers above each box indicate the means of days to flowering for the three growing conditions (2013XN = at year 2013, Qinghai, spring environment; 2014LZ = at year 2014, Gansu, spring environment; 2014WH = at year 2014, Wuhan, semi-winter environment). **, significant difference according to *t*-test (*α* = 0.05), *P* < 0.01. ‘mHAP’ marker-based haplotype. (TIFF 554 kb)

Additional file 9: Variance of phenotype for flowering time of all the transgenic lines harboring different *BnaA3.FRI* haplotypes and Col-0. (A) The phenotype at flowering stage, (B) days to flowering; (C) rosette leaf numbers at bolting stage. Letters indicate significant differences according to *t*-test (*α* = 0.05) (TIFF 1290 kb)

Additional file 10: Multiple sequence alignment between *BnaC3.FRI* and *BolC.FRI.a*. The Genbank accession numbers of *BolC.FRI.a*-E1 and *BolC.FRI.a*-E8 alleles used for the alignment were XP_013629499.1 and AFB73850.1, respectively. Stars indicate the non-synonymous mutations. Arrows indicate the two locations of amino acid deletions. (TIFF 6910 kb)

## Abbreviations

BAC: bacterial artificial chromosome
CFP: mTurquoise2
CO: CONSTANS
FRI: FRIGIDA
FT: FLOWERING LOCUS T
GUS: β-glucuronidase
HAP: Haplotype
INDEL: Insertion/deletion
LFY: LEAFY
mHAP: Haplotype based on marker
QTL: Quantitative trait locus
SNP: Single nucleotide polymorphisms
SOC1: SUPPRESSOR OF OVEREXPRESSION OF CONSTANS
SOR: Spring oilseed rape
SWOR: Semi-winter oilseed rape
UTR: Untranslated regions
WOR: Winter oilseed rape
YFP: mVenus

## References

[1] Fornara F, de Montaigu A, Coupland G. SnapShot: Control of flowering in Arabidopsis. Cell. 2010;141(3):550, 550 e551-552. 10.1016/j.cell.2010.04.024 20434991

[2] Boss PK, Bastow RM, Mylne JS, Dean C (2004). Multiple pathways in the decision to flower: enabling, promoting, and resetting. Plant Cell.

[3] Putterill J, Laurie R, Macknight R (2004). It's time to flower: the genetic control of flowering time. BioEssays.

[4] Moon J, Lee H, Kim M, Lee I (2005). Analysis of flowering pathway integrators in Arabidopsis. Plant Cell Physiol.

[5] Jaeger KE, Wigge PA (2007). FT protein acts as a long-range signal in Arabidopsis. Curr Biol.

[6] Wigge PA (2011). FT, a mobile developmental signal in plants. Curr Biol.

[7] Richard MA, Michaels SD (2010). The timing of flowering. Plant Physiol.

[8] Michaels SD, Amasino RM (1999). FLOWERING LOCUS C encodes a novel MADS domain protein that acts as a repressor of flowering. Plant Cell.

[9] Michaels SD, Amasino RM (2001). Loss of FLOWERING LOCUS C activity eliminates the late-flowering phenotype of FRIGIDA and autonomous pathway mutations but not responsiveness to vernalization. Plant Cell.

[10] Shindo C, Aranzana MJ, Lister C, Baxter C, Nicholls C, Nordborg M, Dean C (2005). Role of FRIGIDA and FLOWERING LOCUS C in determining variation in flowering time of Arabidopsis. Plant Physiol.

[11] Choi K, Kim J, Hwang HJ, Kim S, Park C, Kim SY, Lee I (2011). The FRIGIDA complex activates transcription of FLC, a strong flowering repressor in Arabidopsis, by recruiting chromatin modification factors. Plant Cell.

[12] Hepworth SR, Valverde F, Ravenscroft D, Mouradov A, Coupland G (2002). Antagonistic regulation of flowering-time gene SOC1 by CONSTANS and FLC via separate promoter motifs. EMBO J.

[13] Hu X, Kong X, Wang C, Ma L, Zhao J, Wei J, Zhang X, Loake GJ, Zhang T, Huang J (2014). Proteasome-mediated degradation of FRIGIDA modulates flowering time in Arabidopsis during vernalization. Plant Cell.

[14] Bastow R, Mylne JS, Lister C, Lippman Z, Martienssen RA, Dean C (2004). Vernalization requires epigenetic silencing of FLC by histone methylation. Nature.

[15] Johanson U (2000). Molecular analysis of FRIGIDA, a major determinant of natural variation in Arabidopsis flowering time. Science.

[16] Stinchcombe JR, Weinig C, Ungerer M, Olsen KM, Mays C, Halldorsdottir SS, Purugganan MD, Schmitt J (2004). A latitudinal cline in flowering time in Arabidopsis Thaliana modulated by the flowering time gene FRIGIDA. Proc Natl Acad Sci U S A.

[17] Chalhoub B, Denoeud F, Liu S, Parkin IA, Tang H, Wang X, Chiquet J, Belcram H, Tong C, Samans B (2014). Plant genetics. Early allopolyploid evolution in the post-Neolithic Brassica Napus oilseed genome. Science.

[18] Long Y, Shi J, Qiu D, Li R, Zhang C, Wang J, Hou J, Zhao J, Shi L, Park BS (2007). Flowering time quantitative trait loci analysis of oilseed brassica in multiple environments and genomewide alignment with Arabidopsis. Genetics.

[19] Cai CC, Tu JX, Fu TD, Chen BY (2008). The genetic basis of flowering time and photoperiod sensitivity in rapeseed (Brassica Napus L.). Genetika.

[20] Raman H, Raman R, Eckermann P, Coombes N, Manoli S, Zou X, Edwards D, Meng J, Prangnell R, Stiller J (2013). Genetic and physical mapping of flowering time loci in canola (Brassica Napus L.). Theor Appl Genet.

[21] Wang N, Chen B, Xu K, Gao G, Li F, Qiao J, Yan G, Li J, Li H, Wu X (2016). Association mapping of flowering time QTLs and insight into their contributions to rapeseed growth habits. Frontiers in Plant Science..

[22] Xu L, Hu K, Zhang Z, Guan C, Chen S, Hua W, Li J, Wen J, Yi B, Shen J (2016). Genome-wide association study reveals the genetic architecture of flowering time in rapeseed (Brassica Napus L.). DNA Res.

[23] Axeisson T, Shavorskaya O, Lagercrantz U (2001). Multiple flowering time QTLs within several brassica species could be the result of duplicated copies of one ancestral gene. Genome.

[24] Tadege M, Sheldon CC, Helliwell CA, Stoutjesdijk P, Dennis ES, Peacock WJ (2001). Control of flowering time by FLC orthologues in Brassica Napus. Plant J.

[25] Hou J, Long Y, Raman H, Zou X, Wang J, Dai S, Xiao Q, Li C, Fan L, Liu B (2012). A tourist-like MITE insertion in the upstream region of the BnFLC.A10 gene is associated with vernalization requirement in rapeseed (Brassica Napus L.). BMC Plant Biol.

[26] Osborn TC, Kole C, Parkin IA, Sharpe AG, Kuiper M, Lydiate DJ, Trick M (1997). Comparison of flowering time genes in Brassica Rapa, B. Napus and Arabidopsis Thaliana. Genetics.

[27] Schiessl S, Iniguez-Luy F, Qian W, Snowdon RJ (2015). Diverse regulatory factors associate with flowering time and yield responses in winter-type Brassica Napus. BMC Genomics.

[28] Wang N, Qian W, Suppanz I, Wei L, Mao B, Long Y, Meng J, Muller AE, Jung C (2011). Flowering time variation in oilseed rape (Brassica Napus L.) is associated with allelic variation in the FRIGIDA homologue BnaA.FRI.a. J Exp Bot.

[29] Doyle JDJ (1987). A rapid DNA isolation procedure for small quantities of fresh leaf tissue. Phytochem Bull.

[30] Gookin TE, Assmann SM (2014). Significant reduction of BiFC non-specific assembly facilitates in planta assessment of heterotrimeric G-protein interactors. Plant J.

[31] Xue W, Xing Y, Weng X, Zhao Y, Tang W, Wang L, Zhou H, Yu S, Xu C, Li X (2008). Natural variation in Ghd7 is an important regulator of heading date and yield potential in rice. Nat Genet.

[32] Clough SJ, Bent AF (1998). Floral dip: a simplified method for agrobacterium-mediated transformation ofArabidopsis thaliana. Plant J.

[33] Yang H, Liu J, Huang S, Guo T, Deng L, Hua W (2014). Selection and evaluation of novel reference genes for quantitative reverse transcription PCR (qRT-PCR) based on genome and transcriptome data in Brassica Napus L. Gene.

[34] Jefferson RA, Kavanagh TA, Bevan MW (1987). GUS fusions: β-glucuronidase as a sensitive and versaltile gene fusion marker in higher plants. EMBO J.

[35] Voinnet O, Rivas S, Mestre P, Baulcombe D (2003). An enhanced transient expression system in plants based on suppression of gene silencing by the p19 protein of tomato bushy stunt virus. Plant J.

[36] Risk JM, Laurie RE, Macknight RC, Day CL (2010). FRIGIDA and related proteins have a conserved central domain and family specific N- and C- terminal regions that are functionally important. Plant Mol Biol.

[37] Schiessl S, Samans B, Huttel B, Reinhard R, Snowdon RJ (2014). Capturing sequence variation among flowering-time regulatory gene homologs in the allopolyploid crop species Brassica napus. Front Plant Sci.

[38] Irwin JA, Lister C, Soumpourou E, Zhang Y, Howell EC, Teakle G, Dean C (2012). Functional alleles of the flowering time regulator FRIGIDA in the Brassica oleracea genome. BMC Plant Biol.

[39] Gazzani S, Gendall AR, Lister C, Dean C (2003). Analysis of the molecular basis of flowering time variation in Arabidopsis accessions. Plant Physiol.

[40] Le Corre V, Roux F, Reboud X (2002). DNA polymorphism at the FRIGIDA gene in Arabidopsis Thaliana: extensive nonsynonymous variation is consistent with local selection for flowering time. Mol Biol Evol.

