# Supplementary figures and images for: Sequence variation and functional analysis of a FRIGIDA orthologue (BnaA3.FRI) in Brassica napus
Source: BMC Plant Biol. 2018 Feb 13;18:32. doi: 10.1186/s12870-018-1253-1 (PMC5810009; doi:10.1186/s12870-018-1253-1)

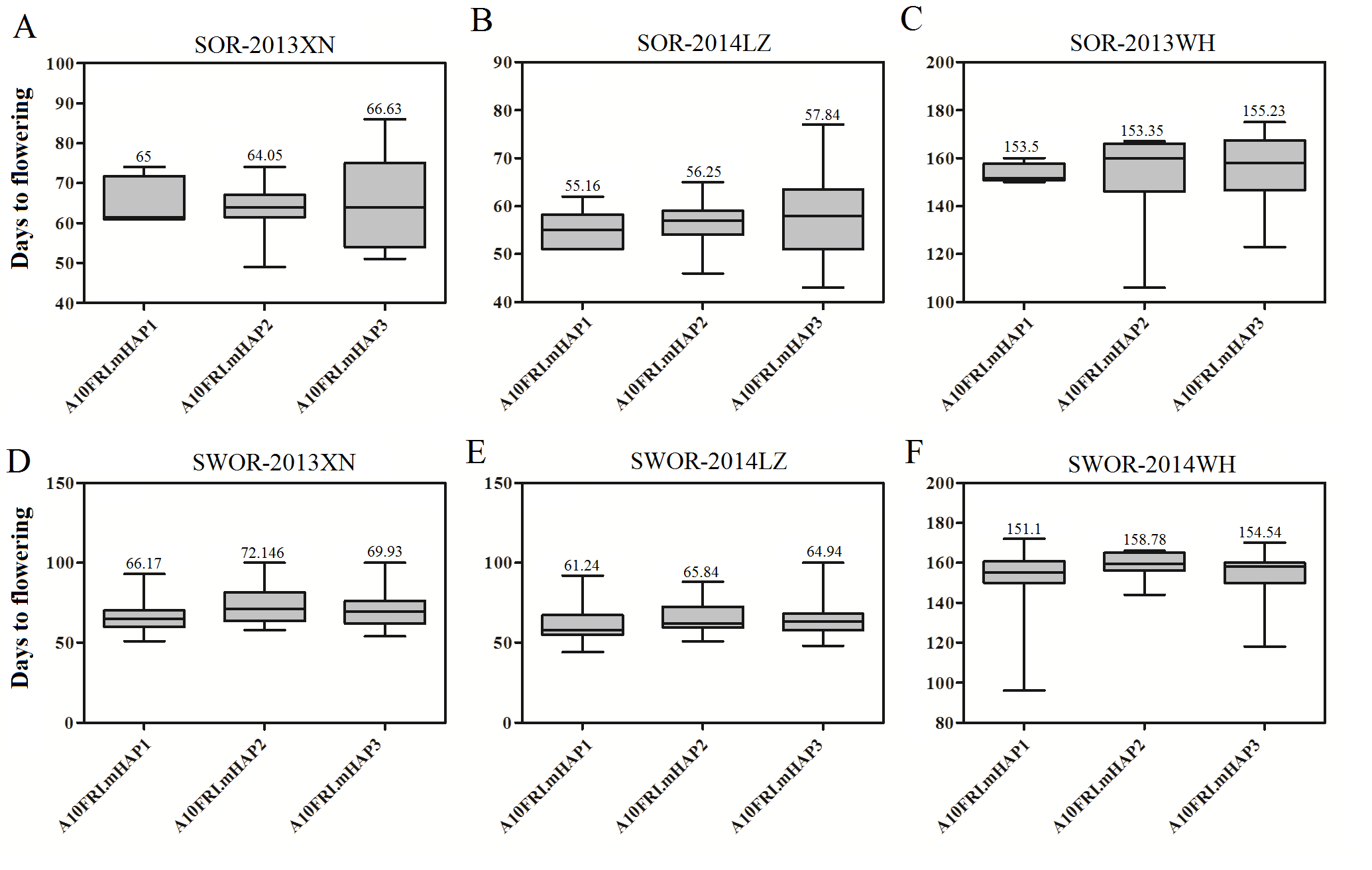

Supplement: Supplementary file 6 — The effect of different BnaA10.FRI haplotypes on mean days to flowering in SORs (accession numbers of mHAP1-3 were 6, 20, and 13, respectively) (A-C) and SWORs (accession numbers of mHAP1-3 were 69, 14, and 35, respectively) (D-F). Numbers above each box indicate the means of days to flowering for the three growing conditions (2013XN = at year 2013, Qinghai, spring environment; 2014LZ = at year 2014, Gansu, spring environment; 2014WH = at year 2014, Wuhan, semi-winter environment). ‘mHAP’ marker-based haplotype. (TIFF 421 kb) [file 12870_2018_1253_MOESM6_ESM.tif]

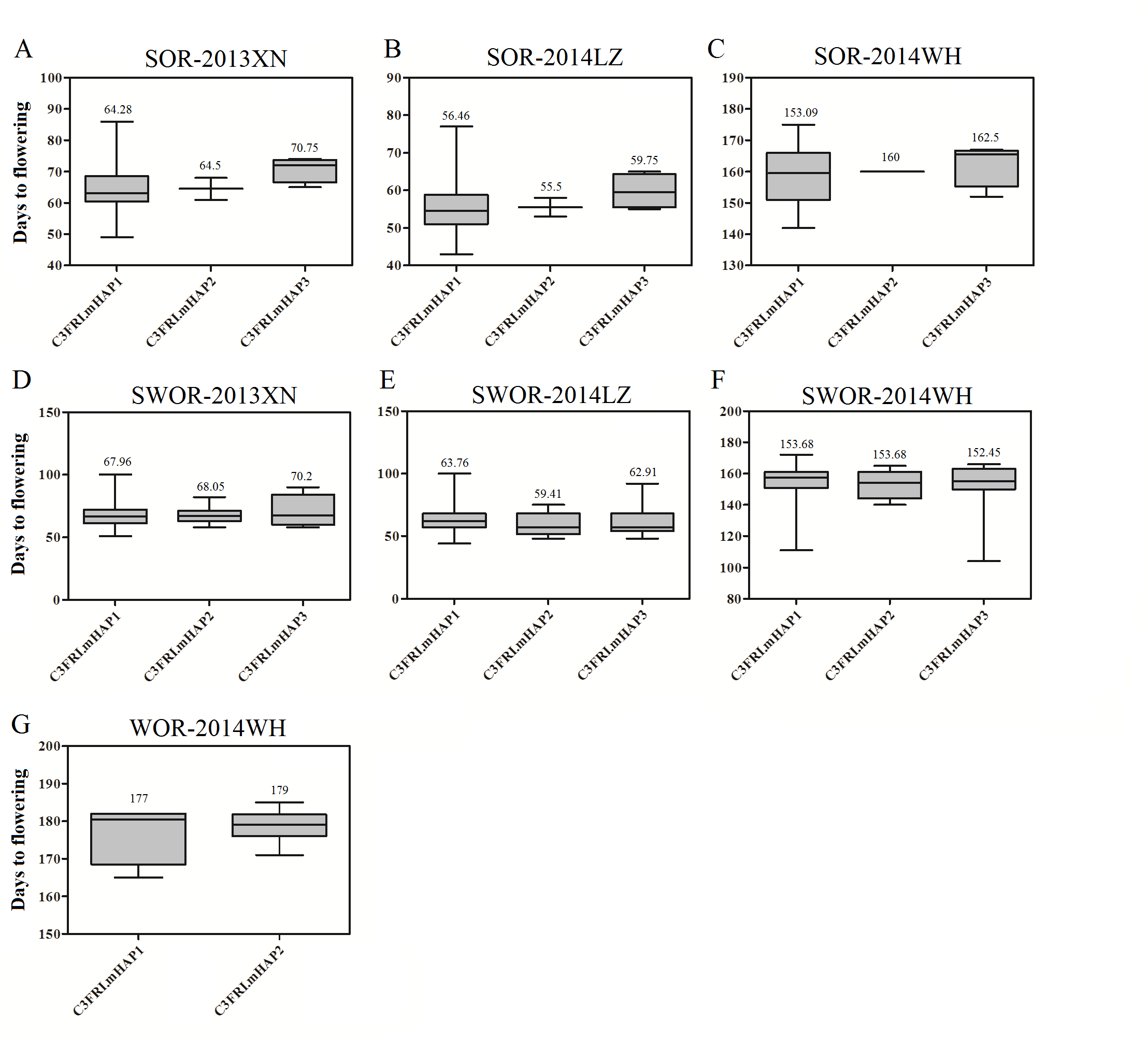

Supplement: Supplementary file 7 — The effect of different BnaC3.FRI haplotypes on mean days to flowering in SORs (accession numbers of mHAP1-3 were 33, 2, and 4, respectively) (A-C), and SWORs (accession numbers of mHAP1-3 were 87, 19, and 11, respectively) (D-F), and WOR (accession numbers of mHAP1 and 2 were 5 and 12, respectively) (G). Numbers above each box indicate the means of days to flowering for the three growing conditions (2013XN = at year 2013, Qinghai, spring environment; 2014LZ = at year 2014, Gansu, spring environment; 2014WH = at year 2014, Wuhan, semi-winter environment). ‘mHAP’ marker-based haplotype. (TIFF 590 kb) [file 12870_2018_1253_MOESM7_ESM.tif]

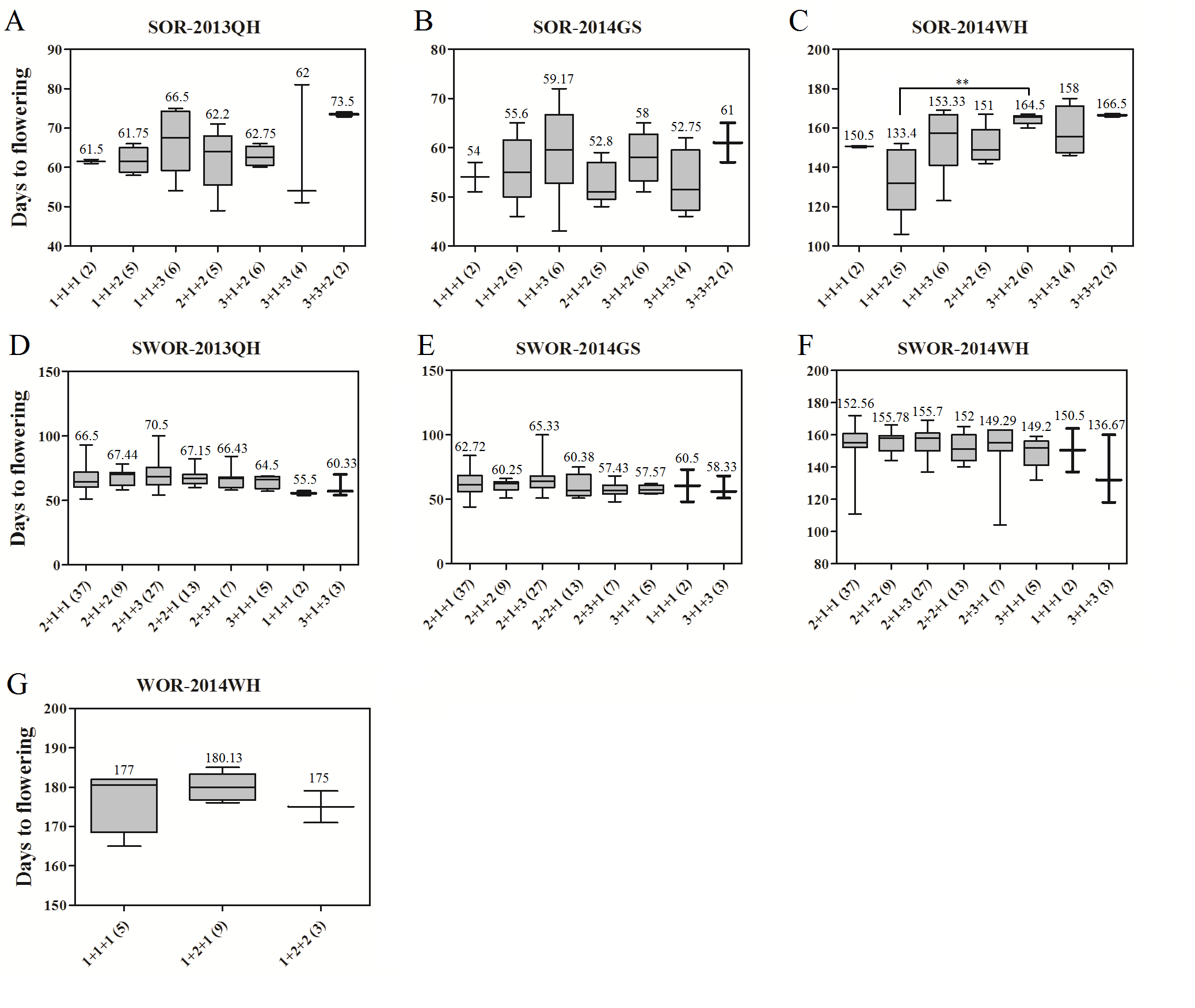

Supplement: Supplementary file 8 — The effect of combinations of BnaFRIs haplotypes on mean days to flowering in SORs (A-C), SWORs (D-F), and WOR (G). 1 + 1 + 1 indicates the combinations of BnaA3.FRI + BnaC3.FRI + BnaA10.FRI, and so on for the rest. Numbers in brackets indicate the accession number of each combination. Numbers above each box indicate the means of days to flowering for the three growing conditions (2013XN = at year 2013, Qinghai, spring environment; 2014LZ = at year 2014, Gansu, spring environment; 2014WH = at year 2014, Wuhan, semi-winter environment). **, significant difference according to t-test (α = 0.05), P < 0.01. ‘mHAP’ marker-based haplotype. (TIFF 554 kb) [file 12870_2018_1253_MOESM8_ESM.tif]

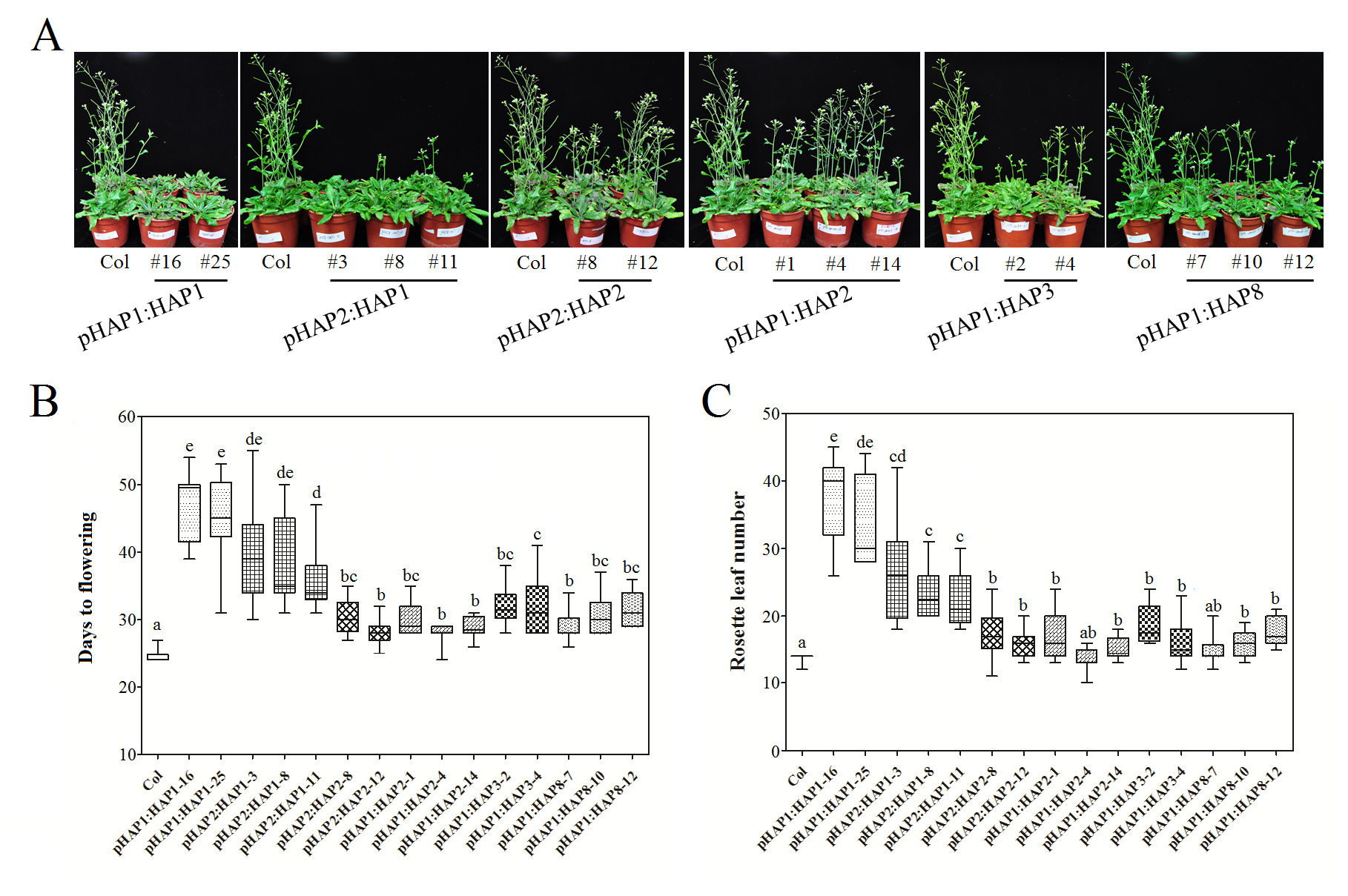

Supplement: Supplementary file 9 — Variance of phenotype for flowering time of all the transgenic lines harboring different BnaA3.FRI haplotypes and Col-0. (A) The phenotype at flowering stage, (B) days to flowering; (C) rosette leaf numbers at bolting stage. Letters indicate significant differences according to t-test (α = 0.05) (TIFF 1290 kb) [file 12870_2018_1253_MOESM9_ESM.tif]

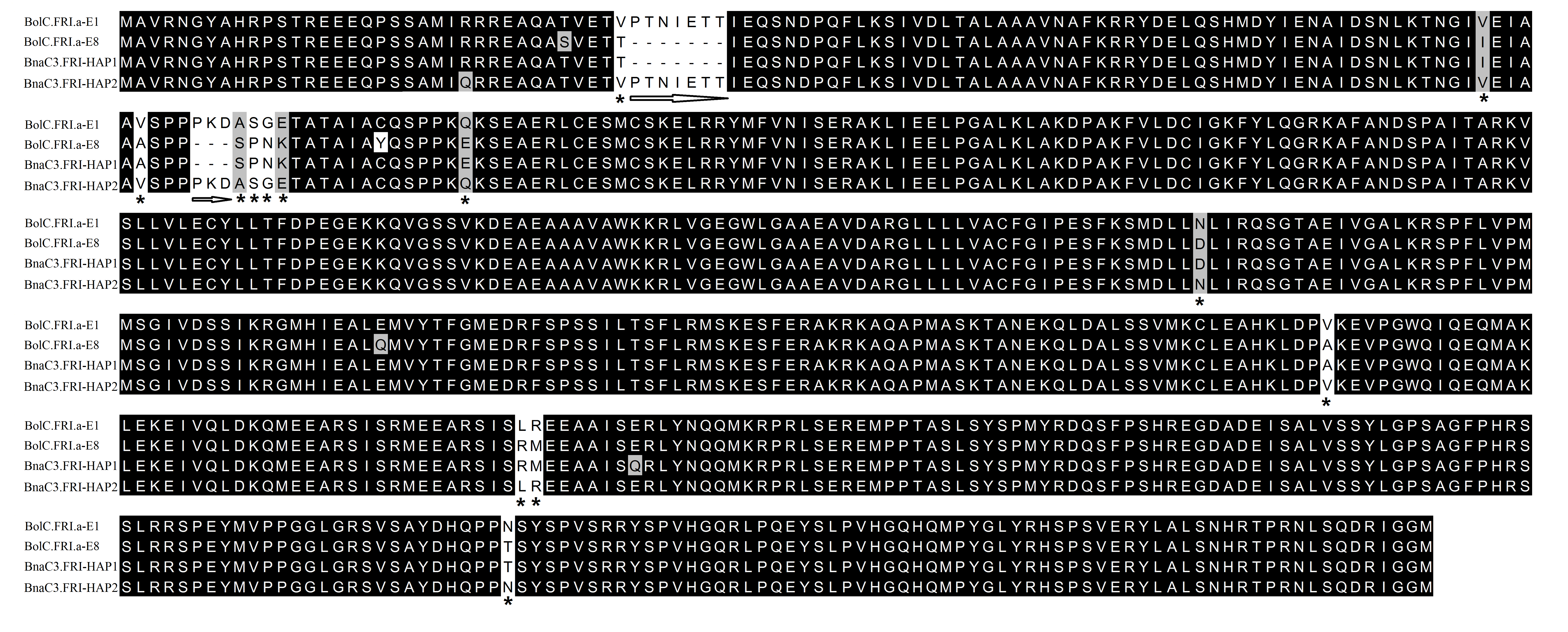

Supplement: Supplementary file 10 — Multiple sequence alignment between BnaC3.FRI and BolC.FRI.a. The Genbank accession numbers of BolC.FRI.a-E1 and BolC.FRI.a-E8 alleles used for the alignment were XP_013629499.1 and AFB73850.1, respectively. Stars indicate the non-synonymous mutations. Arrows indicate the two locations of amino acid deletions. (TIFF 6910 kb) [file 12870_2018_1253_MOESM10_ESM.tif]
